# Neural Correlates of Inhibitory Control in Children: Evidence Using MRI and fNIRS

**DOI:** 10.1007/s10548-025-01129-8

**Published:** 2025-07-26

**Authors:** Leela Shah, Xin Zhou, Marissa Ann DiPiero, Jayse Merle Weaver, Corrina Frye, Steven R. Kecskemeti, Ruth Y. Litovsky, Andrew L. Alexander, Elizabeth M. Planalp, Douglas C. Dean

**Affiliations:** 1 Waisman Center, University of Wisconsin–Madison, 1500 Highland Ave, Madison, WI 53705 USA; 2 Neuroscience Training Program, University of Wisconsin-Madison, 1111 Highland Ave, Madison, WI 53705 USA; 3 Brain and Mind Institute, The Chinese University of Hong Kong, 4 F, Hui Yeung Shing Building, N.T, Hong Kong, Hong Kong; 4 Medical Physics, University of Wisconsin–Madison, 1111 Highland Ave #1005, Madison, WI 53705 USA; 5 Division of Otolaryngology, Department of Surgery, University of Wisconsin-Madison, 600 Highland Ave, Madison, WI 53792 USA; 6 Psychiatry, University of Wisconsin–Madison, 6001 Research Park Blvd, Madison, WI 53719 USA; 7 Pediatrics, University of Wisconsin-Madison, 600 Highland Ave, Madison, WI 53792 USA

**Keywords:** Functional near-infrared spectroscopy, Quantitative relaxometry, Magnetic resonance imaging, Inhibitory control, Brain activation, Myelin

## Abstract

Inhibitory control (IC) develops in stages from infancy through adolescence and is associated with numerous developmental disorders and learning outcomes. This study examined how neural architecture – in particular myelination – underlies brain activation patterns observed during IC tasks in a sample of 28 children aged 4–10 years old. IC was observed using reaction times during go/no-go and flanker IC tasks. Myelination was measured using quantitative longitudinal relaxation rate (R1) mapping obtained from selected white matter regions of interest (ROIs). Brain activation was defined as task-related changes in hemoglobin oxygenation as measured by functional near-infrared spectroscopy (fNIRS) averaged within ROIs. Results indicated that myelination in ROIs was higher in older children and fNIRS activation in frontal channels was significantly and positively associated with go/no-go mean reaction time. Myelination in the corona radiata and superior longitudinal fasciculus was positively associated with frontal fNIRS activation, while myelination was negatively associated with go/no-go and flanker mean reaction times across white matter ROIs. Overall, significance level notably varied across models. Independently of inhibitory control constructs, these regions may be of interest in future structure-function studies across development.

**Supplementary Information:**

The online version contains supplementary material available at 10.1007/s10548-025-01129-8.

## Highlights

We measured myelination, brain activation, and inhibitory control task reaction times.

Myelination in white matter regions was associated with task reaction times.

Frontal cortical brain activation was associated task reaction times.

Myelination may support the relationship between brain activation and reaction times.

**Supplementary Information:**

The online version contains supplementary material available at 10.1007/s10548-025-01129-8.

## Introduction

Inhibitory control (IC) is the ability to manage and suppress behaviors, emotions, and actions to achieve behavioral or cognitive goals (Diamond 2013). This executive function is essential in everyday tasks, such as controlling impulses, making decisions, and adapting to new environments (Gilbert and Burgess 2008; Kusi-Mensah et al. 2022). IC is one of the earliest higher order cognitive functions to emerge, yet it has a prolonged developmental trajectory, with basic motor response IC beginning to develop in infancy (Kochanska et al. 1998) and more complex cognitive IC develops from middle childhood through adolescence (Deshaies and Éthier 2024; Ordaz et al. 2013; Simpson and Carroll 2019). Deficits in IC are implicated in impulsivity, distractibility, and aggressiveness, as well as in conditions such as oppositional defiant disorder and conduct disorder (Bonham et al. 2021), autism spectrum disorder (Schmitt et al. 2018) and attention-deficit/hyperactivity disorder (ADHD, Bonham et al. 2021; Elosúa et al. 2017; Schachar et al. 1995). Since IC spans motor, emotional, verbal, and cognitive domains, studying its development and impacts on later pathology is an important area of research (Deshaies and Éthier 2024).

Insights from functional (fMRI), structural, and diffusion (dMRI) imaging studies have contributed to the current understanding of brain regions and networks involved in IC development and expression. fMRI studies in children and adults demonstrate increased activation with age during IC tasks in the inferior frontal gyrus, insula, orbitofrontal gyrus, lingual gyrus, and anterior cingulate gyrus (Cohen et al. 2010; Cope et al. 2020; Ordaz et al. 2013; Rubia et al. 2006, 2007; Tamm et al. 2002), with one study reporting higher activation in frontal and striatal regions in females and parietal regions in males (Rubia et al. 2013). Electroencephalography (EEG) studies demonstrate changes in frontal EEG power during IC tasks in preschool-aged children that improve longitudinally with improved task performance. These include larger N2 component amplitudes associated with go/no-go task training (Liu et al. 2015), lower lateral frontal power associated with maternal-report measures of inhibitory control (Morasch and Bell 2011), and larger baseline-to-task changes in medial frontal EEG activity associated with improved task accuracy (Watson and Bell 2013). fNIRS studies in preschool children demonstrate associations between prefrontal cortex activation and more irritability during IC tasks (Fishburn et al. 2019; Li et al. 2016), and in children and adults demonstrate age-based differences in connectivity patterns during a go/no-go task, including reduced inhibition-related right fronto-parietal activation in children compared to adults (Mehnert et al. 2013). More recently, we also observed higher fNIRS cortical activity of the right prefrontal cortex and left orbitofrontal cortex to be associated with better performance and smaller standard error of mean of reaction time in IC tasks in children (Zhou et al. 2022). Taken together, these studies highlight that as individuals age, better performance during IC tasks is linked to specific neural patterns, including higher frontal activation, changes in EEG power, and stronger connectivity.

Structural MRI studies have further implicated the dorsolateral prefrontal cortex, inferior frontal gyri, anterior cingulate cortex, basal ganglia, subthalamic nucleus, hippocampus, and fornix in IC (Benear et al. 2020; Godsil et al. 2013; Munakata et al. 2011; Ou et al. 2023; Pérez-Cervera et al. 2023). Additional dMRI work has revealed microstructural correlates of IC in typical development (Goddings et al. 2021a) and ADHD (Tremblay et al. 2020), with lower frontostriatal radial diffusivity associated with better IC (Liston et al. 2006). Other dMRI studies have associated higher fractional anisotropy in the anterior corona radiata, right inferior frontal gyrus, and right presupplementary motor cortex and lower radial diffusivity in the right inferior frontal gyrus and right presupplementary with improved IC (Mackiewicz Seghete et al. 2013; Madsen et al. 2010). These structural findings suggest myelin maturation, which enhances neural processing and communication, may have a significant role in the development of IC. Indeed, myelination has been shown to underlie differences in functional brain activation and executive function ability, including IC ability (Fiske and Holmboe 2019; Fornari et al. 2007; Goddings et al. 2021a, b; Ribeiro et al. 2024).

As the aforementioned studies have demonstrated numerous brain regions involved in IC, it is likely that the development of IC requires a large-scale network with no single dominant brain region (Kang et al. 2022). Moreover, it is unclear how brain structure relates to brain activation in regions across these networks during development, and how these associations ultimately relate to IC. One multimodal study found age-related decreases in insular and middle frontal gyrus volume and activation on fMRI, which related to slower response time in a go/no-go task (Hu et al. 2018). Additional work related gray matter volume and fNIRS prefrontal activation during a dual task involving cognitive load and walking task in elderly individuals, suggesting an important association between brain structure and task-based activation (Wagshul et al. 2019).

IC development has been studied using a range of experimental paradigms, including the go/no-go, stop-signal, flanker, Stroop, and antisaccade tasks (Friedman and Robbins 2022). Many of these tasks have been adapted to preschool and early elementary-aged children by incorporating age-appropriate tasks that similarly assess these skills (Isquith et al. 2005). While the go/no-go task requires inhibiting a motor response, the flanker task requires inhibiting attention towards irrelevant stimuli (Friedman and Robbins 2022). In elementary-aged children, IC task accuracies are uniformly high; thus, reaction times are used to capture variability in IC in this population (Schulz et al. 2023; Ursache and Raver 2014). Reaction times tend to be slower (or longer) during tasks that require inhibition of a response, and become faster (shorter) with improvements in task accuracy and overall fluid intelligence (Schulz et al. 2023). Further, higher brain activation as measured by fNIRS and fMRI is associated with IC performance and faster reaction times (Cohen et al. 2010; Zhou et al. 2022). However, to the best of our knowledge, no multimodal study exists that connects brain myelination patterns that may underlie hand task-related brain activation during IC in children, leaving questions of how brain structure and brain activation relate to IC ability, and how these relations differ at points across development.

In this study, we leverage a multi-modal approach to understanding the dynamic interplay between brain structure, activation, and behavior across sensitive periods of childhood. We evaluate how myelination across early to middle childhood relates to task-related functional brain activation and how these structural and functional neuroimaging features correlate with neurobehavioral assessments of IC. We evaluate associations between myelination as measured by quantitative relaxometry (R1) acquired using the MPnRAGE technique (Kecskemeti et al. 2016; Kecskemeti and Alexander 2020) in awake children, fNIRS oxygenated hemoglobin levels measured during a go/no-go task, and behavioral results from the go/no-go task and the flanker test of IC from the NIH Toolbox (Weintraub et al. 2013), in a sample of children aged 4–10 years. For both the go/no-go task and the flanker task assessments, we operationalized IC performance using reaction times (RTs). We chose R1, the inverse of T1 relaxation time, to quantify myelination given its sensitivity in quantifying myelin content across development (Deoni et al. 2012; Kühne et al. 2021). Finally, we used fNIRS to quantify brain activation given its improved temporal sensitivity over fMRI to blood flow changes related to cortical neuronal activation and ability to be used in a more natural environment than fMRI (Fishburn et al. 2014; Pinti et al. 2015).

Our hypotheses were as follows: (1) Similar to previous work, we anticipate age-related and sex-related differences in R1, task-related fNIRS oxygenated hemoglobin changes, and IC performance (Cope et al. 2020; de Rooij and Weeda 2020; Deoni et al. 2012; Fornari et al. 2007; Ho et al. 2024; Rubia et al. 2013; Wu et al. 2025; Zhou et al. 2022); (2) Based on prior work relating fNIRS oxygenated hemoglobin to IC ability during the go/no-go task (Zhou et al. 2022), higher oxygenated hemoglobin levels during the portions of the go/no-go task requiring IC will be related to faster RTs during both the go/no-go and flanker tasks; (3) Larger R1 will be associated with faster RTs on both IC tasks, as R1 is sensitive to increased myelination, which may facilitate improved processing speed (Dean et al. 2015); and (4) R1 will be positively associated with fNIRS oxygenated hemoglobin levels, based on prior work correlating myelination with higher brain activation in adults (Fornari et al. 2007; Huang et al. 2023). Additionally, as prior literature has examined myelination and brain activation relating to IC ability (Dean et al. 2015; Fornari et al. 2007; Huang et al. 2023; Zhou et al. 2022), we explored whether R1 moderated the relationship between fNIRS oxygenated hemoglobin levels and IC ability, such that higher myelin content (larger R1) would result in a stronger relationship between brain activation and IC.

## Materials and Methods

### Participants

This study included behavioral, MRI and fNIRS data collected from 51 children, aged 4–11 years old. Children were recruited from Waisman Center recruitment registries and via mass email distributed to university employees and students. Children underwent fNIRS, MRI, and behavioral testing on the same day. Inclusion criteria were lack of contraindications to MRI scanning, lack of limitations due to physical health, typical development, and English as primary language. Exclusion criteria included diagnosis of psychiatric or neurologic illness, developmental disorders, inability to undergo MRI scanning, or mothers with medical conditions or significant illness during pregnancy. Participants with incomplete MRI acquisition or fNIRS acquisition were excluded from the study, resulting in a sample of 28 children (14 females) between 4 and 10 years of age (mean = 6.8 years, SD = 1.9 years). Demographics are included in Table 1; sample age (full mean = 6.7 years, full SD = 2.5 years) and sex (full sample = 19 females) were not significantly different between the full and limited samples (*p* >.05) as assessed by t-test. Experimental protocols were approved by the University of Institutional Review Board and written informed consent was provided by the parent or primary caregiver for each child with verbal assent provided by each child.

**Table 1:** Sample demographic characteristics

Variables	Sample (n)
Number of subjects	28
Mean age (years)	6.77
*Child Sex*	
Male	14
Female	14
*Child Race (incl. multiple)*	
White	21
Asian	2
American Indian/AK Native	1
Other	1
Not provided	5
Hispanic/Latino (ethnicity)	2

### Data Acquisition and Processing

Data acquisition and processing methods are described in the ensuing paragraphs; the behavioral and fNIRS data acquisition methods are also described in (Zhou et al. 2022). The data analysis and processing methods are dated to (Zhou et al. 2020).

### Multimodal Go/no-Go Task

Children completed a go/no-go task in a sound-treated booth, seated 1.5 m from a computer monitor. Images were presented at the center, right, or left side of the monitor. Sounds were presented at a comfortable level from three loudspeakers (Tannoy Reveal 402) located in front of (0° azimuth), on the right (40° azimuth), or to the left (320° azimuth) of the child.

There were two types of trials administered during the go/no-go task:

### Congruent Trial

At the beginning of the task, a dog image was shown at the center of the monitor, followed by a “bark” sound played by the center speaker after a 0.35 s delay. The dog then disappeared over a duration of 2.35 s. Children were trained to respond within 2 s after the onset of the sound and before the disappearance of the dog by pressing a one-button computer mouse when seeing a dog and hearing a “bark”. Between trials, a red balloon appeared on the left or right side of the monitor for 0.6s, to indicate the location for the ensuing images and sounds. After the balloon cue, a dog image was shown and a “bark” played from the balloon-indicated location – this trial was known as a “congruent” trial as the sound and image corresponded to the same animal and were in the same location as previously indicated by the red balloon.

### Incongruent Trial

There were two types of incongruent trials. For type 1 incongruent trials, the child might see a dog image but hear a “meow” sound, or vice versa. For type 2 incongruent trials, the balloon clue was on the incorrect side as the subsequent animal image/noise combination. Children were trained not to respond to incongruent sounds and images. Type 2 incongruent trials were introduced to add extra interference between informative cues and subsequent events. We did not distinguish the two types of incongruent trials in the data analyses, as they both were aimed to require IC through differential activation or suppression of motor responses depending on task requirements.

“Go” events refer to congruent sounds and images, and “NoGo” events refer to incongruent sounds and images. Response to a “Go” event and lack of response to a “NoGo” event were met with a smiley face appearing on the monitor for 0.6s.

Children were trained on the rules of the task at their own pace before data collection. Once the experimenter was confident that the child was familiar with the rules, the child was taken to the fNIRS booth for another practice session, consisting of three congruent blocks and two incongruent blocks, at the same pace as the actual testing, but with no fNIRS data recorded. Throughout learning, practicing, and testing, the ratio of congruent trials to incongruent trials was approximately 2.5:1.

**Fig. 1:**
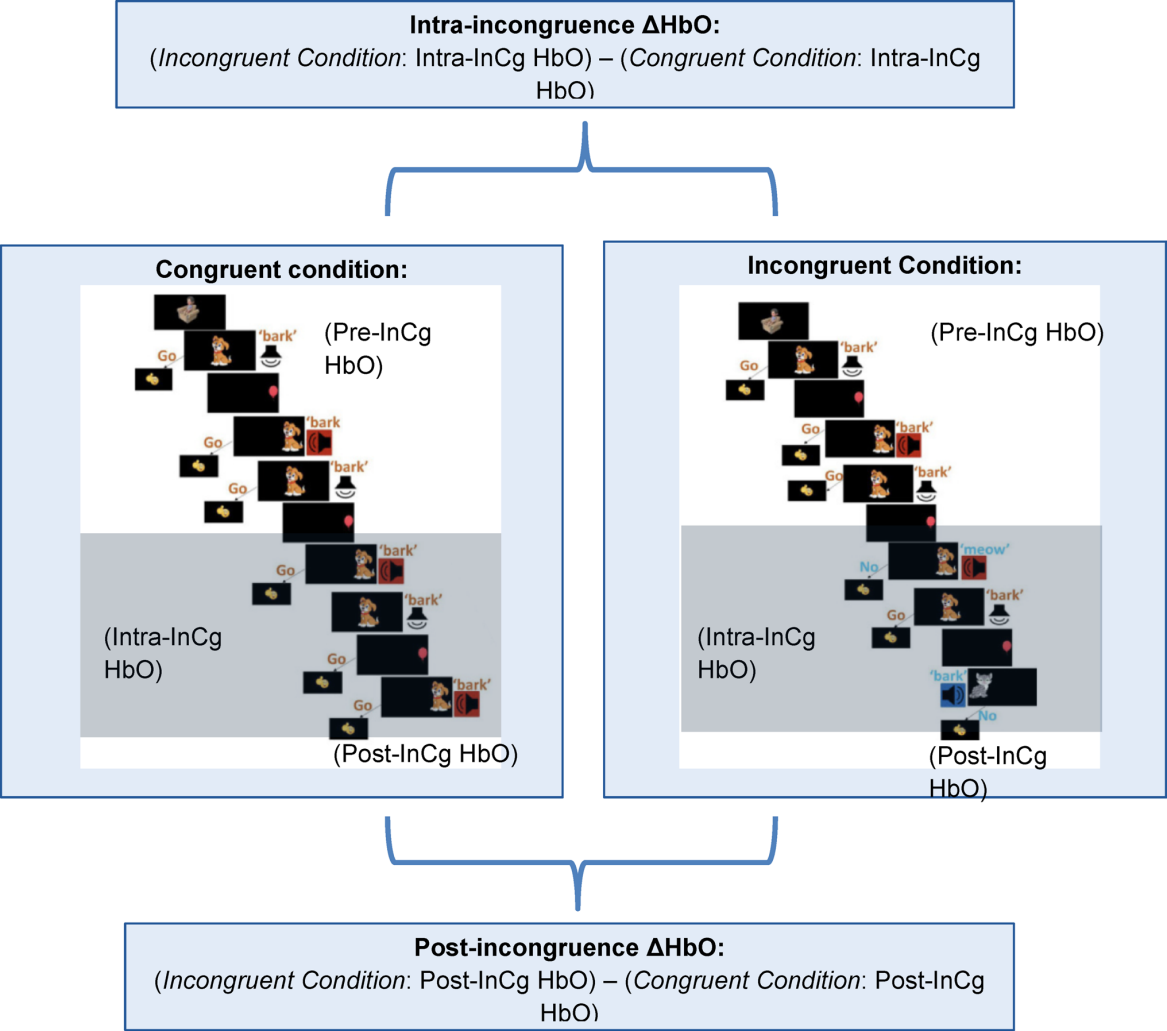
Trial design for congruent (Cg) and incongruent (InCg) conditions, along with calculation of Intra-incongruence ΔHbO and post-incongruence ΔHbO from HbO measured at three time points (pre-InCg, intra-InCg, and post-InCg) during Cg and InCg conditions. For each condition, the pre-InCg HbO measurement was the average HbO from 0 to 10.7 s following stimulus onset, the intra-InCg HbO measurement was the average HbO from 10.7 to 20.7 s following stimulus onset, and the post-InCg HbO measurement was after stimulus offset, and was the average HbO from 20.7 to 30 s following stimulus onset. Intra-incongruence ΔHbO was calculated by subtracting intra-InCg HbO between the and Cg and InCg conditions, and post-InCg ΔHbO was calculated by subtracting post-Incg HbO between the InCg and Cg conditions

The overall block design consisted of two conditions. A congruent block (20.7 s) consisted of three congruent trials, while an incongruent block (20.7 s) consisted of one congruent trial, followed by two incongruent trials. The order of the type of incongruent trials was randomized across blocks. In the same block, the same dog image was presented, but across blocks, four different dog images and four different cat images were used to minimize boredom effects. After the onset of each block, there was a 12-s baseline where children were seeing four different and irrelevant cartoon pictures. The congruent condition was the grand mean average of all 8 congruent blocks, while the incongruent condition was the grand mean average of all 8 incongruent blocks. Blocks were administered across two 5-minute periods of 8 blocks each. See Fig. 1 for a breakdown of conditions.

The difference in mean RT between the incongruent and congruent conditions was calculated for all participants for the go/no-go task, in order to determine the effect of IC (required by the incongruent trials) on reaction times (Zhou et al. 2022). A faster mean RT indicates that children were faster at responding to task stimuli, representing better IC.

### NIH Toolbox Flanker Task

Participants underwent a modified version of the flanker task as part of the NIH toolbox cognition battery, which is further described in (Zelazo et al. 2013). Using an iPad, participants were presented with a central stimulus surrounded by congruent or incongruent stimuli (flankers) and were asked to indicate the left-right orientation of the central stimulus. In congruent trials, the orientation of the flanking stimuli matched the orientation of the central stimulus, while the orientation of the flanking stimuli was opposite to the central stimulus in incongruent trials.

Both scaled score and mean RT across all trials were calculated from the flanker assessment, but analyses were nonsignificant with scaled score, so only mean RT (flanker mean RT) are included in the current analyses. The test administration did not have separate incongruent and congruent trials as in the go/no-go task, so flanker mean RT was calculated as the overall mean RT of the task.

### Functional Near-infrared Spectroscopy Data Acquisition

fNIRS data were collected in a sound-treated booth via a continuous-wave NIRScout system (NIRx, Medical Technologies, LLC). The 16 light sources and 16 avalanche photodiode detectors were arranged to cover the frontal and temporal cortex in both hemispheres, and their orientation is pictured in (Zhou et al. 2022). Data acquisition and signal processing techniques are further described in (Zhou et al. 2020). For children with dense hair, hairpins were used to improve scalp exposure to avoid impacting light intensity. Caps were readjusted with the consent of the child to maximize light intensity.

### Functional Near-infrared Spectroscopy Data Analysis

We determined differences in task-related brain activation as measured by fNIRS task-related changes in oxygenated hemoglobin (HbO). HbO levels were measured during three time points for each condition (congruent and incongruent, Fig. 1). For the congruent condition, all 3 trials were congruent trials. For the incongruent condition, the second 1.5 trials were both incongruent trials. The pre-incongruence (pre-InCg) HbO was calculated from the first 1.5 trials (within the first 10.7 s post stimulus onset) as this was the time period in both incongruent and congruent conditions when a subject could only be exposed to congruent trials, prior to the introduction of incongruent trials. The intra-incongruence (intra-InCg) HbO timepoint for each condition was the grand mean HbO across the second 1.5 trials (10.7–20.7 s after stimulus onset), beginning with the first incongruent event. The post-incongruence (post-InCg) HbO timepoint for each condition was the period (20.7 to 30 s after stimulus onset) following the third trial. This duration was chosen to match the duration of the pre-incongruence and intra-incongruence period.

Intra-incongruence ΔHbO was the difference in intra-InCg HbO between the incongruent and congruent conditions. This was the difference in brain activation between the incongruent condition, which required IC, and the congruent condition, which did not require IC. Higher values indicate higher brain activation during trials which require IC. Post-incongruence ΔHbO was the difference in post-InCg HbO between the incongruent and congruent conditions. This was the difference in brain activation following completion of the incongruent versus congruent conditions.

**Fig. 2:**
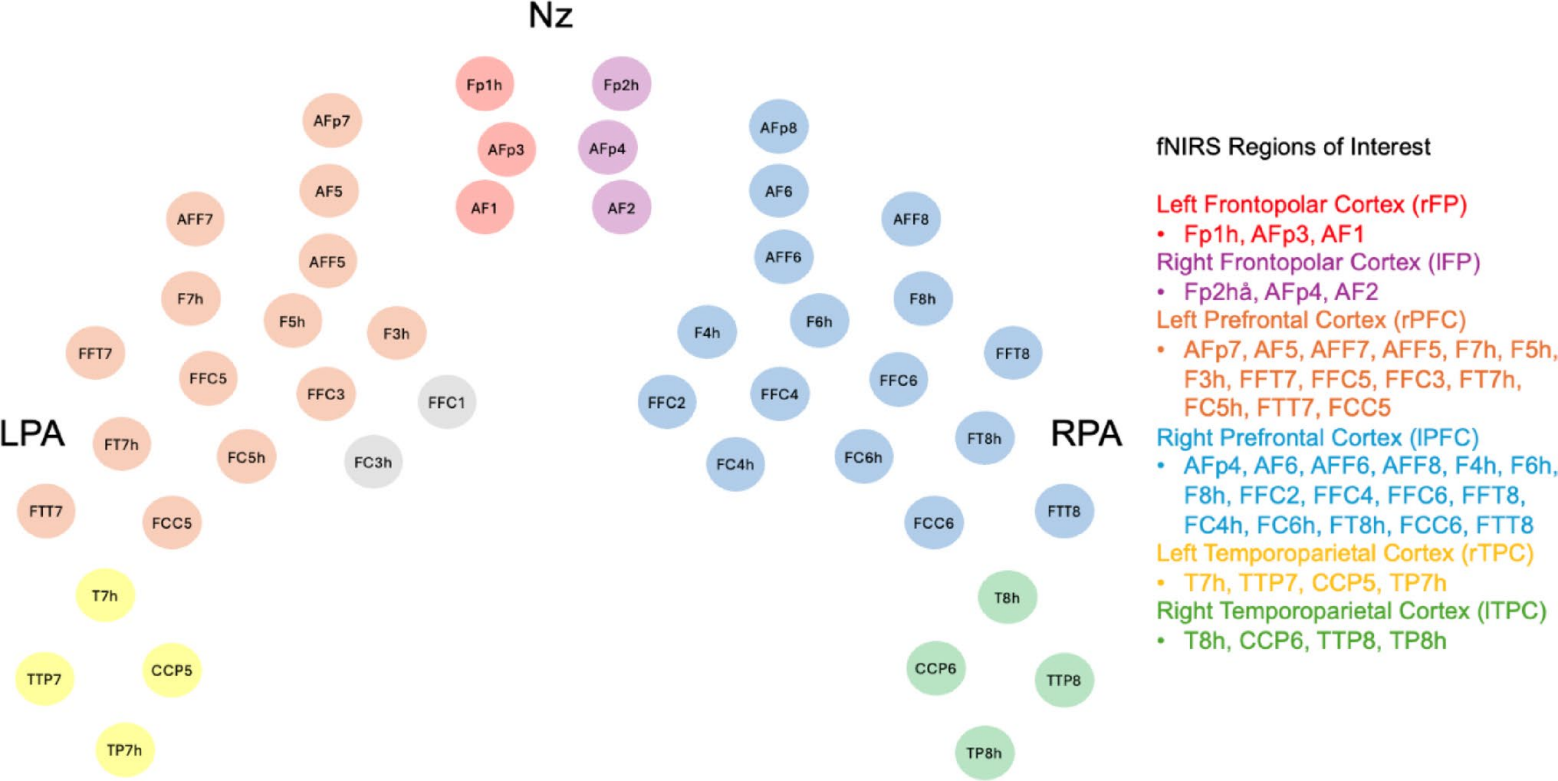
Functional near-infrared spectroscopy, spatial representation of channels. Labels are included to orient the location of channels, with Nz representing the nasion (anterior), and RPA and LPA representing the right and left preauricular points, respectively. The 46 fNIRS channels are shown, with the color-coded legend on the right representing the 6 ROIs from which mean ΔHbO was calculated. This figure was adapted from NIRSite software (NIRSite 2.0, NIRx Medical Technologies, LLC), as well as from (Zhou et al. 2022) with the authors’ permission. Channels in gray (FFC1 and FC3h) were excluded from analysis given poor data quality

As described in (Zhou et al. 2022), we chose to analyze oxygenated hemoglobin changes (ΔHbO) as opposed to deoxygenated hemoglobin changes (ΔHbR) as previous work has suggested that, while the two values are negatively correlated, ΔHbO measurements exhibit larger amplitudes of change and improved signal-to-noise ratios (Bright 2012; Cui et al. 2010; Hoshi 2016; Quaresima and Ferrari 2019). Regions of interest (ROIs) were calculated as ΔHbO averaged across clusters of optodes, which covered the right frontal pole (rFP), left frontal pole (lFP), right prefrontal cortex (rPFC), left prefrontal cortex (lPFC), and right temporoparietal cortex (rTPC), left temporoparietal cortex (lTPC), as outlined in Fig. 2.

### MRI Data Acquisition

MRI data were acquired using a 3 T GE Discovery MR750 scanner (Waukesha, WI) with a 32-channel phased array head coil (Nova Medical, Wilmington, MA). Whole-brain structural imaging was performed using a 3D T1-weighted MPnRAGE sequence with 1 mm isotropic resolution and an inversion-recovery magnetization preparation and three-dimensional radial *k*-space acquisition (Kecskemeti et al. 2016). Acquisition parameters included TR = 4.9 ms, TE = 1.8 ms, and 386 views along the recovery curve, with excitation flip angles of 4° for the first 304 views and 8°for the 82 remaining views. A delay time of TD = 500 ms occurred after the last TR of each gradient-echo block to allow the signal to recover before the next preparation pulse. Scan duration was approximately 8 min. All participants watched a video of their choice during scanning and were instructed to remain still. Retrospective motion correction was performed as described in (Kecskemeti et al. 2018).

### MRI Data Analysis

R1 was used to quantify myelination within selected white matter regions across the brain. A multi-pass fitting procedure was used to estimate parametric maps of T1 relaxation times and denoised using total variation minimization (Kecskemeti and Alexander 2020). Age and study-specific templates were created from participants’ MPnRAGE T1-weighted images using the “antsMultivariateTemplateConstruction2.sh” script as part of ANTs (Avants et al. 2014). Individual participants T1 maps were transformed to the template space from the resulting affine and non-linear transformations. The Johns Hopkins University (JHU) ICBM-DTI-81 template (Hua et al. 2008; Mori et al. 2005; Wakana et al. 2007) was spatially aligned to the population template using ANTs (Avants et al. 2014). The population-template aligned JHU ICBM-DTI-81 white matter atlas was then transformed to each subject’s native space by applying the inverse of the spatial transformations estimated in the population- and subject-specific template generation step. Median values were extracted from each participant’s corresponding native-space T1 map. Median values were used as the median is less sensitive to voxels with extreme values (Van Belle et al. 2004). R1 values were calculated as the inverse of T1 relaxation times, to allow for ease of comparison. Higher R1 values indicate higher myelin content.

White matter ROIs were limited to regions thought to be involved in IC functions, including the genu of the corpus callosum (GCC), body of the corpus callosum (BCC), splenium of the corpus callosum (SCC), fornix (F), right and left anterior corona radiata (r/lACR), right and left superior corona radiata (r/lSCR), right and left posterior corona radiata (r/lSCR), right and left cingulate gyrus (r/lCG), right and left cingulum (hippocampal aspect, r/lCH, right and left fornix segments (rF/lF), right and left superior longitudinal fasciculus (rSLF/lSLF), right and left superior fronto-occipital fasciculus (r/lSFOF), right and left inferior fronto-occipital fasciculus (r/lIFOF), and right and left uncinate fasciculus (r/lUF).

### Visit Timeline

Children were tested with the NIH toolbox assessments, fNIRS go/no-go task, and MRI in separate sessions on the same day. The order of the three tests varied based on family preference and scheduling needs, with MRI generally collected last. NIH toolbox assessments were administered in a quiet playroom. fNIRS data were acquired during the go/no-go task in a sound-treated booth.

### Statistical Analyses

First, we log-transformed flanker and go/no-go mean RT to address general linear model violations of normality as assessed using the GVLMA package in R (Pena and Slate 2019). All further analyses use the log-transformed values. Before conducting our focal analyses, we addressed hypothesis 1 by examining simple correlations among all variables with age and sex to determine if these relationships aligned with those reported in prior literature (Deoni et al. 2012; Ho et al. 2024).

To address our 3 focal hypotheses, we conducted a series of general linear models in R, with log(age) and sex and their interactions included, as sex differences in brain-behavior relationships may be contingent on age, which is not addressed using simple correlations (DeCasien et al. 2022). For each model, non-significant effects of age and sex (either direct effect or interaction) were removed if model fit was improved. Model fit was assessed using Akaike’s Information Criterion (AIC), where a change in AIC greater than 2 between two comparison models indicates that the model with a lower AIC has superior model fit (Burnham and Anderson 2004). False discovery rate (FDR) was used to adjust p-values for multiple comparisons, correcting for the number of brain regions examined with each model (Benjamini and Hochberg 1995).

Specific analyses to address Hypotheses 2–4, following adjustments to improve model fit, are as follows: To assess hypothesis 2, we examined associations between ΔHbO and mean RT to evaluate how ΔHbO during the parts of the task requiring IC related to RTs on that task. Separate models were conducted for intra-incongruence and post-incongruence ΔHbO as well as both flanker and go/no-go tasks.

$$\hbox{Hypothesis 2 Model:}\:Mean\:RT\:={\beta\:}_{0}+\:\:{\beta\:}_{1}*\varDelta\:HbO+\:{\beta\:}_{2}*AGE+\epsilon\:$$

For hypothesis 3, we examined associations between R1 and mean RT values to evaluate the relationship between myelination and task performance. Again, separate models were conducted for both the flanker and go/no-go tasks.

$$\hbox{Hypothesis 3 Model}:\:Mean\:RT\:={\beta\:}_{0}+\:\:{\beta\:}_{1}*R1+\:{\beta\:}_{2}*AGE+\epsilon\:$$

Hypothesis 4 asked whether myelin content was related to task-related brain activation. We examined whether R1 was associated with intra-incongruence or post-incongruence ΔHbO in any of the predefined ROIs.

$$\hbox{Hypothesis 4 Model:}\:\varDelta\:HbO\:={\beta\:}_{0}+\:\:{\beta\:}_{1}*R1+\:{\beta\:}_{2}*AGE+\epsilon\:$$

Lastly, in a more exploratory analysis building on analyses 2–4, only in regions where intra-incongruent or post-incongruent ΔHbO and R1 were associated with go/no-go scores, we examined whether R1 and ΔHbO uniquely or synergistically related to IC go/no-go task performance.

$$\begin{aligned} Mean~RT~~ =& \beta _{0} + ~\beta _{1} *R1 + ~\beta _{2} *\Delta HbO \\ & + ~\beta _{3} *\Delta HbO*R1 + \beta _{4} *AGE + \varepsilon ~ \\ \end{aligned}$$

## Results

### Age-Related Changes in Myelin, Task-Related Brain Activation, and IC

**Fig. 3:**
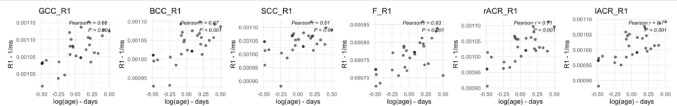
Scatter plots of log(age) in days and average R1 in selected regions in 1/milliseconds (1/ms). Pearson correlation r values and corresponding p values are included for each selected region. Regions include: the genu of the corpus callosum (GCC), body of the corpus callosum (BCC), splenium of the corpus callosum (SCC), fornix (F), right anterior corona radiata (rACR), and left anterior corona radiata (rACR)

There were no significant correlations between sex and log(age), flanker RT, or go/no-go RT (all *p* >.05). There were also no significant correlations between sex and R1, intra-incongruence ΔHbO, or post-incongruence ΔHbO in our regions of interest (all *p* >.05).The correlation between R1 and log(age) was significant (*p* <.05) in all regions except the right fornix (Fig. 3). Intra-incongruence and post-incongruence ΔHbO were not significantly associated with age in any ROIs. Flanker mean RT was significantly correlated with log(age), both before (*p* <.0001) and after (*p* <.0001) log transformation. Go/no-go mean RT was not significantly correlated with age.

*.*

### Hypothesis 2 Reaction Time and Brain Activation

**Fig. 4:**
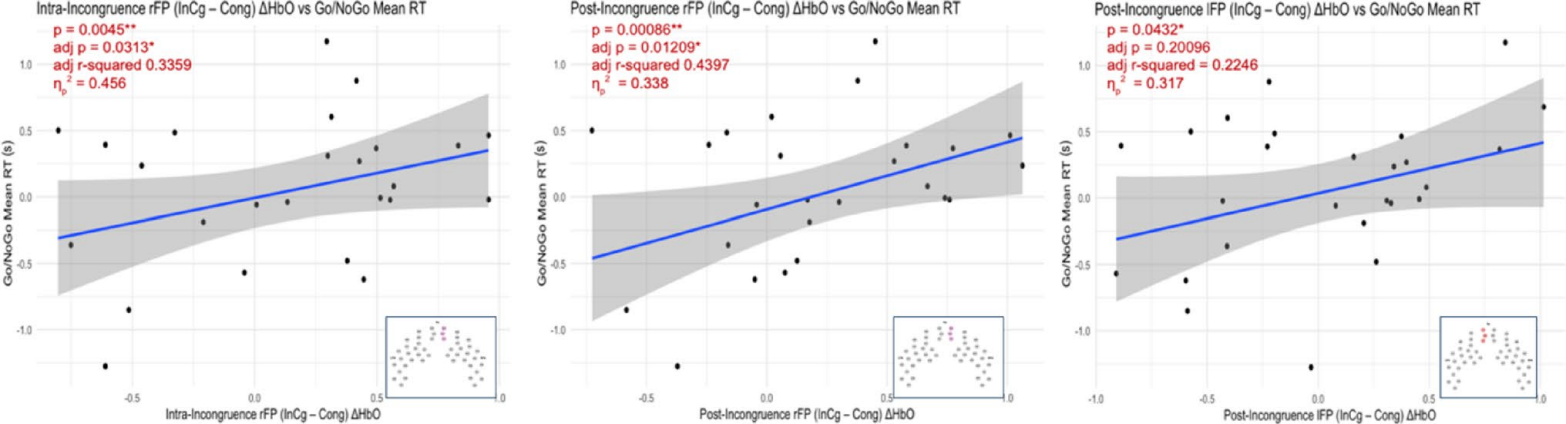
Relationships between intra-incongruence or post-incongruence ΔHbO and go/no-go mean RT. The covariate and interaction terms for sex and interaction terms for age were not significant and did not improve model fit, so were removed

Task-related change in fNIRS brain activation in the right frontopolar and left frontopolar region was associated with go/no-go mean RT. Specifically, intra-incongruence ΔHbO in the rFP (*p* =.005, adj *p* =.031) and post-incongruence ΔHbO in the rFP (*p* =.001, adj *p* =.012) were significantly positively associated with go/no-go mean RT. All other associations were non-significant (adj *p* >.05), although a relationship emerged between post-incongruence ΔHbO in the lFP that did not pass tests for multiple comparisons (*p* =.043, adj *p* =.201). Effect sizes (partial eta squared - η_p_^2^) for these relationships were on the order of 0.32 to 0.46, indicating large effects. Log(age) was significant in these models (*p* <.05), with a larger effect of ΔHbO in older children than younger children in models; sex was not significant as a covariate or interaction term and age was not significant as an interaction term; these predictors were therefore removed to improve model fit assessed by AIC (Fig. 4, Burnham and Anderson 2004). Intra-incongruence and post-incongruence ΔHbO were not significantly associated with flanker mean RT in any of the regions analyzed.

*.*

### Hypothesis 3: Reaction Time and Myelination

**Fig. 5:**
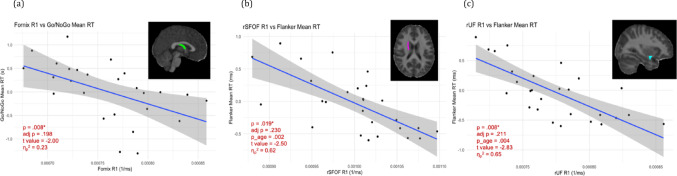
Relations between go/no-go or flanker mean RT and R1 in the Fornix (panel A), right superior fronto-occipital fasciculus (rSFOF; panel B), and right uncinate fasciculus (rUF, panel C). Log(age) was significant in the models associated with flanker mean RT (B&C) and was removed from all additional models

No significant relationships between R1 values in our ROIs and flanker mean RT emerged after multiple comparisons corrections (all adj *p* >.05). Nonetheless, some preliminary relationships emerged that may inform future analyses. R1 in the fornix was negatively associated with go/no-go mean RT, such that larger R1 (higher myelination) predicted faster reaction times (*p* =.008, adj *p* =.198, η_p_^2^ = 0.23). In the rSFOF and rUF, R1 was negatively associated with flanker mean RT (rSFOF: *p* =.019, adj *p* =.230; rUF: *p* =.008, adj *p* =.211). Log(age) was significant in two models (rSFOF: *p* =.002; rUF: *p* =.004), with a stronger effect of R1 on flanker mean RT in older children (Fig. 5).

### Hypothesis 4: Myelination and Brain Activation

**Fig. 6:**
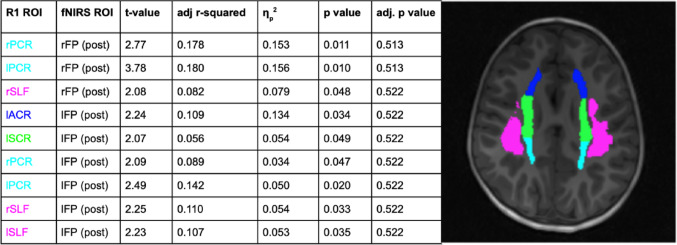
Relationships between R1 and intra-incongruence rFP, post-incongruence rFP, and post-incongruence lFP ΔHbO. Each row represents a model with R1 in the ROIs labeled (R1 ROI) modeled with the outcome variable ΔHbO in the ROI labeled (fNIRS ROI), with all relationships including post-incongruence ΔHbO. R1 ROIs include: the right posterior corona radiata (rPCR), left posterior corona radiata (lPCR), right superior longitudinal fasciculus (rSLF), left anterior corona radiata (lACR), and left superior corona. fNIRS RO! s include the right fronto-polar region (rFP) and left fronto-polar region (lFP)

Given the relationship observed between bilateral frontal activation and go/no-go mean RT, we examined relationships between R1 in all white matter ROIs and frontal intra-incongruence and post-incongruence ΔHbO. No significant relationships between R1 in white matter ROIs and ΔHbO values emerged after multiple comparisons corrections (all adj *p* >.05, η_p_^2^: 0.03 to 0.15). We have included a summary of preliminary analyses which may inform future work (Fig. 6).

### Exploratory Post-hoc Analysis: Go/no-go Reaction Time and the Interaction of Myelination and Brain Activation

Given the posited role of myelination in underlying brain activation changes (Fornari et al. 2007), we conducted exploratory analyses assessing an interactive relationship between myelination and brain activation on go/no-go mean RT. We focused on go/no-go mean RT given that flanker mean RT was not associated with intra-incongruence or post-incongruence ΔHbO in any regions. Focusing on pairs of regions where R1 and ΔHbO were individually associated with go/no-go mean RT, we conducted general linear models examining the interactive association between R1 in white matter ROIs and post-incongruence ΔHbO in optode clusters on go/no-go mean RT as the outcome variable, controlling for age. Covariates and interaction terms for sex and interaction terms for age were nonsignificant and excluded to improve model fit assessed by AIC. There was a nonsignificant interaction in the model examining the joint effects of R1 in the fornix and post-incongruence ΔHbO in the rFP on go/no-go mean RT (*p* =.09).

## Discussion

In this work, we use multi-modal neuroimaging and behavioral assessment to investigate the contribution of brain structure and activation to IC ability across childhood. We explore relationships between myelin content (R1), task-related brain activation (fNIRS ΔHbO), and measures of IC performance in a developmentally diverse cohort.

Relations between myelination and IC measures, age, and sex revealed trajectories similar to previous work, with measures of myelin increasing logarithmically with age across the investigated epoch of childhood (Fornari et al. 2007; Ho et al. 2024; Kang et al. 2022). Sex was not significantly correlated with any of our measures and was not significant in our models as a covariate or interaction term. While prior work has established sex-specific brain activation patterns in adolescents and adults in relation to IC (Rubia et al. 2013) and general sex-specific differences in IC performance in children (Silverman 2021), our lack of sex-related findings may be related to sample size, differences in task requirements between our study and others, or the age of our sample.

Task-related brain activation was not significantly associated with age in any of the ROIs identified for the fNIRS measures. This lack of association aligns with prior work with the same dataset suggesting a non-uniform relationship between fNIRS activation and age (Zhou et al. 2022); this could relate to our sample age range, which may be outside of the period for significant age-related changes in brain activation in these regions (Mehnert et al. 2013; Zhou et al. 2022). It is also possible that age-related changes in these ROIs were not observed due to the fNIRS method, or that there are age-related changes in other regions that were not interrogated in this investigation. In terms of IC, reaction times on the flanker, but not the go/no-go task, showed associations with age. These two tasks capture distinct but overlapping aspects of IC (Friedman and Robbins 2022); thus, response inhibition required by the go/no-go task may develop through different mechanisms than the interference inhibition required by the flanker task, with different trajectories across age (Deshaies and Éthier 2024; Mullane et al. 2009; Simpson and Carroll 2019; Wang et al. 2024).

The prefrontal and frontal cortex are associated with executive function ability (Casey et al. 1997; Funahashi and Andreau 2013; Rae et al. 2015). Therefore, that we found go/no-go task-related behaviors associated with brain activation in these regions is perhaps unsurprising given that these fNIRS optodes overlie the prefrontal area. Interestingly, this contradicts our expectation that higher activity in these regions would be associated with *faster* go/no-go reaction time, indicating faster processing speed facilitated by higher activation. Instead, higher activity was associated with slower reaction time. A previous study examining IC using a Stroop test showed that slower reaction times related to interference effects of the test were associated with higher prefrontal activation, which was explained as a compensatory mechanism for the increasing demands of the IC task (Schroeter et al. 2004). The higher frontal region brain activity observed in the current study may represent a compensatory activation due to higher task demands. Additionally, given that this relationship was stronger with increasing child age, such compensatory ability might be strengthened as children mature. It was unsurprising that task-related brain activation was not related to Flanker RT (assessed using the NIH toolbox in a separate session) given that fNIRS was only recorded during the go/no-go task, so task-related higher brain activation related to the go/no-go would not necessarily be expected to be associated with variation in Flanker RT.

Additionally, more developed neural architecture as indicated by higher myelin content was associated with IC responses in the fornix, fronto-occipital fasciculus, and uncinate fasciculus. For the flanker test of IC, more myelination in the rSFOF and rUF was related to faster reaction times. While preliminary and not withstanding multiple comparisons corrections, these associations aligned with our prediction that more myelination would be associated with faster processing speed (Scantlebury et al. 2014; Thaler et al. 2021). The regions were located in the fornix, rSFOF, and rUF, regions previously shown to be involved in IC, working memory, and other aspects of executive functioning (Krogsrud et al. 2018; Nomura et al. 2013; Ou et al. 2023; Senova et al. 2020; Tremblay et al. 2020).

The positive relationship between SLF and ACR myelination and higher task-related activation did not withstand multiple comparisons corrections. Nonetheless, this relationship aligned with prior research suggesting a positive relationship between myelination and brain activation during cognitive tasks (Fornari et al. 2007; Huang et al. 2023; Huntenburg et al. 2017). The relationship between myelin content and frontal activation supports the importance of myelination in executive functioning processes (Frye et al. 2010; Loe et al. 2019; Stave et al. 2017; Urger et al. 2015).

There was preliminary evidence for an interaction between fornix myelination and frontal task-related brain activation during the go/no-go task, such that participants with higher myelination in the fornix showed a stronger positive association between frontal brain activation and task performance. Similar to previous research (Fornari et al. 2007; Huang et al. 2023), this suggests that higher myelination may enhance such brain-behavior relationships. The fornix plays a role in working memory and other EF processes, and fornix fimbriae anatomically connect the hippocampus with the prefrontal cortex (Benear et al. 2020; Godsil et al. 2013; Ou et al. 2023; Pérez-Cervera et al. 2023; Senova et al. 2020). While preliminary, a proposed mechanism for the relationship between myelination and brain activation is that myelination increases synapse efficiency and activity (Fornari et al. 2007). Future studies should further evaluate this posited relationship between myelination and functional task-related brain activation through both moderation and mediation models.

### Strengths and Limitations

This is the first study to our knowledge to combine myelin-sensitive quantitative relaxometry with temporally sensitive fNIRS functional activation data to understand the relationship between neural pathways associated with IC in the developing brain. Previous researchers have conducted similar analyses using dMRI metrics; however, dMRI is less specific to myelination than relaxometry based approaches (O’Muircheartaigh et al. 2014; Scantlebury et al. 2014). Nonetheless, interpretation of R1 as a specific measure of myelin content is limited as R1 is also sensitive to other microstructural processes, including water content, edema, and iron content (Deoni 2010; Deoni and Dean 2021). Future studies utilizing complementary approaches, such as multicomponent relaxometry or quantitative magnetization transfer, would be important to disentangle possible confounding influences. It should be noted that our sample size was small due to some subjects not completing both MRI and fNIRS neuroimaging; however, we believe this limitation is mitigated by combining data from these two imaging modalities to provide complimentary information on the constructs of interest. Further, age and sex of the examined study sample did not significantly differ from that of the full dataset of more than 50 children, thus providing additional confidence that our findings would be applicable to a larger study population. We additionally report effect sizes for all models that were significant prior to corrections for multiple comparisons but failed to pass corrections for multiple comparisons. While these effect sizes may be inflated due to the small sample size (Button et al. 2013), we report these findings to guide hypotheses for future studies. Still, future studies examining these relationships in larger sample sizes will be important. Moreover, these data represent a cross-sectional sample of typically developing children, thus limiting variability in behavioral profile; future work should be conducted across neurodevelopmental conditions to allow for more behavioral variability.

This work spans the ages of four to ten years; therefore, we do not have a large group of children representing any one age. However, this also allowed us to observe how the relationship between myelination and brain activation differs between young children and children nearing adolescence. Additionally, our go/no-go task may not have been challenging enough to accurately assess IC ability across our age range (Petersen et al. 2016), as go/no-go RTs were not significantly faster in older children.

One limitation of fNIRS is that it is only able to capture cortical activity and with poorer temporal resolution than other methods such as EEG, thus lacking information about deeper structures with high temporal specificity. Localization of this methodology is therefore poorer in comparison to MRI data; to combat this issue, we averaged activity across groups of optodes rather than analyzing individual optodes (Rahman et al. 2020; Tremblay et al. 2018).

## Conclusion

In this work, we investigated the role of brain myelination in brain activation patterns observed during IC tasks, within a developmentally diverse cohort of children. Lower frontal brain activation and higher myelin content in the fornix, right uncinate fasciculus, and right superior fronto-occipital fasciculus were associated with faster IC reaction times. Myelin across white matter regions was related to higher brain activation, and higher myelin content in the fornix was related to a stronger positive relationship between brain activation and IC, suggesting a possible role of myelin in facilitating cognitive-load related increases in brain activation. Overall, significance level notably varied across models following multiple comparisons corrections, and power was limited by our sample size. Nonetheless, this work lays a foundation for leveraging multimodal imaging techniques to study relationships between brain myelination, activation, and inhibitory control across stages of child development. Future studies should focus on further elucidating these myelin–brain activation relationships across development with larger longitudinal samples.

## Electronic Supplementary Material

Below is the link to the electronic supplementary material.

Supplementary Material 1

Supplementary Material 2

## Data Availability

The data analyzed for this study are available upon request from the corresponding author, as participants did not consent for their data to be shared publicly.

## Imaging modalities leveraged in current study

fNIRS: Functional near-infrared spectroscopy
T1 relaxometry: T1 relaxometry magnetic resonance imaging

## Regions of interest – fNIRS

r/lFP: Right and left frontal pole
r/lPFC: Right and left prefrontal cortex
rTPC: Right and left temporoparietal cortex

## Regions of interest – MRI

GCC: Genu of the corpus callosum
BCC: Body of the corpus callosum
SCC: Splenium of the corpus callosum
F: Fornix
r/lACR: Right and left anterior corona radiata
r/lSCR: Right and left superior corona radiata
r/lSCR: Right and left posterior corona radiata
r/lCG: Right and left cingulate gyrus
r/lCH: Right and left cingulum (hippocampal aspect)
rF/lF: Right and left fornix segments
rSLF/lSLF: Right and left superior longitudinal fasciculus
r/lSFOF: Right and left superior fronto-occipital fasciculus
r/lIFOF: Right and left inferior fronto-occipital fasciculus
r/lUF: Right and left uncinate fasciculus

## Measurements of interest

Go-no/go and flanker mean reaction time: Go/no-go mean RT, flanker mean RT
R1: Inverse of T1 relaxation time
ΔHbO: Oxygenated hemoglobin
Intra-InCg ΔHbO: Oxygenated hemoglobin levels during the go/no-go incongruent condition
Post-InCg ΔHbO: Oxygenated hemoglobin levels following the go/no-go incongruent condition
