# Supplementary material for: Neural Correlates of Inhibitory Control in Children: Evidence Using MRI and fNIRS
Source: Brain Topogr. 2025 Jul 26;38(5):54. doi: 10.1007/s10548-025-01129-8 (PMC12296776; doi:10.1007/s10548-025-01129-8)

**Figure A1**

*Complete collection of scatter plots of log(age) in days and average R1 in all regions in 1/milliseconds (1/ms). Pearson correlation r values and corresponding p values are included for each selected region.*


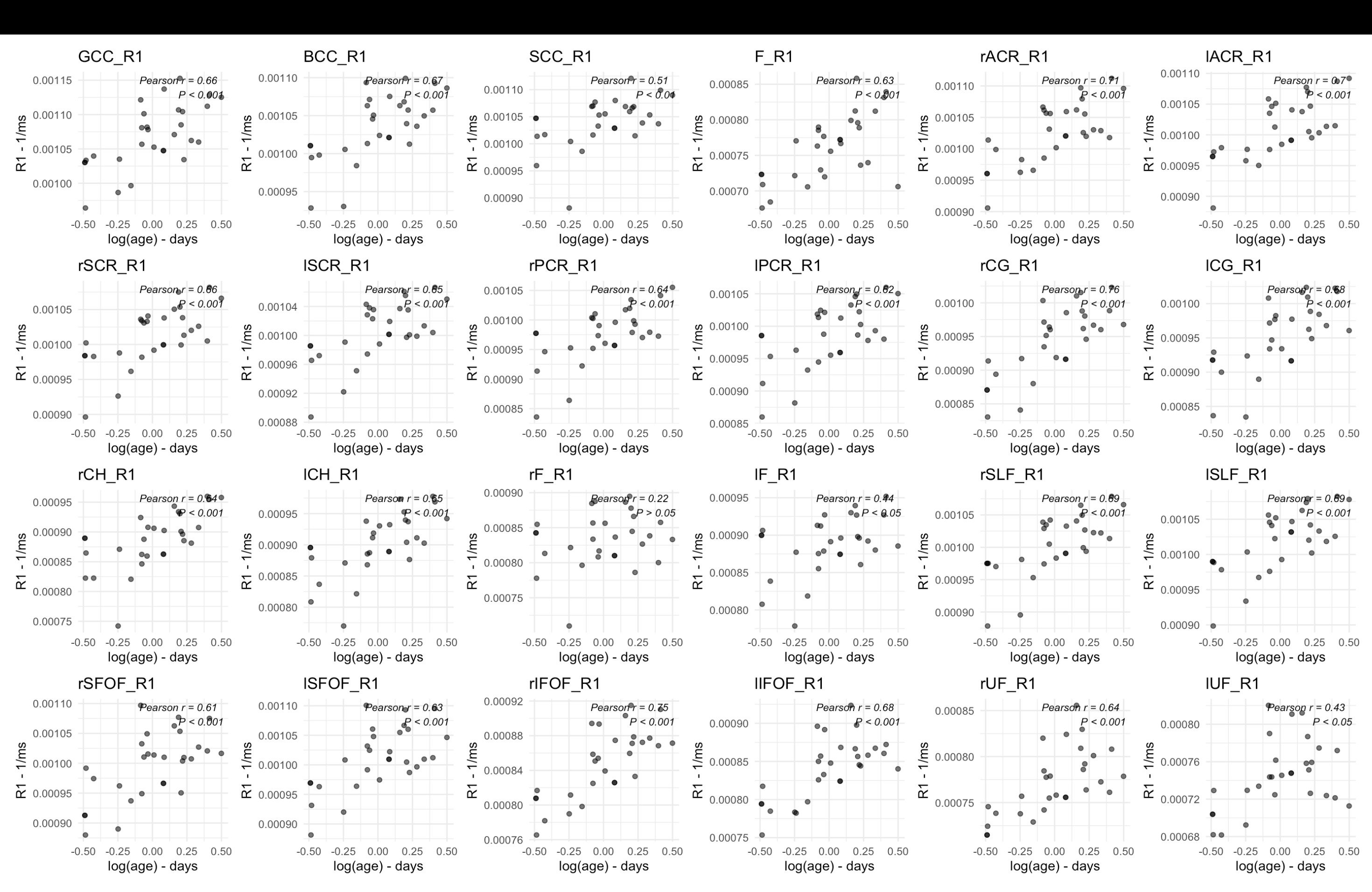

Supplement: Supplementary file 1 — Supplementary Material 1 [file 10548_2025_1129_MOESM1_ESM.docx]
