# Supplementary material for: Neural Correlates of Inhibitory Control in Children: Evidence Using MRI and fNIRS
Source: Brain Topogr. 2025 Jul 26;38(5):54. doi: 10.1007/s10548-025-01129-8 (PMC12296776; doi:10.1007/s10548-025-01129-8)
Supplement: Supplementary file 2 — Supplementary Material 2 [file 10548_2025_1129_MOESM2_ESM.pptx]

## Slide 1
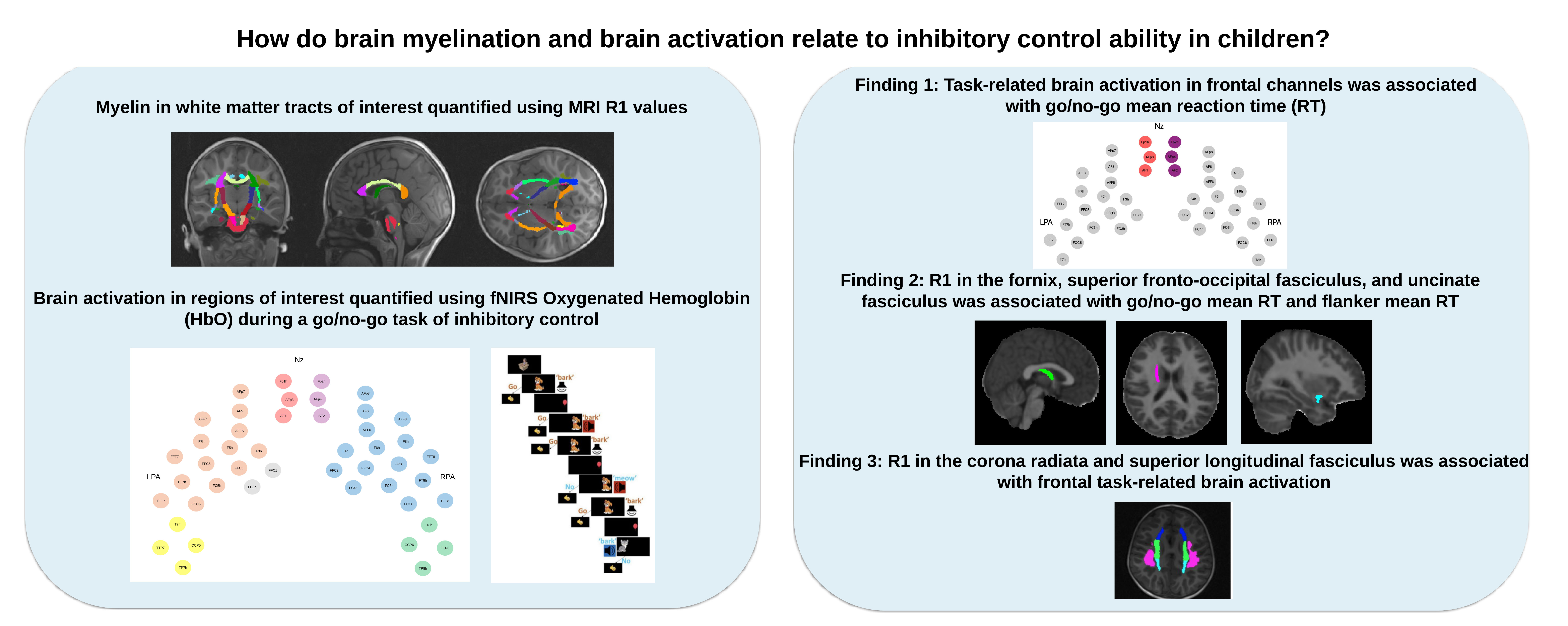

How do brain myelination and brain activation relate to inhibitory control ability in children?
Myelin in white matter tracts of interest quantified using MRI R1 values
Finding 1: Task-related brain activation in frontal channels was associated with go/no-go mean reaction time (RT)
Finding 2: R1 in the fornix, superior fronto-occipital fasciculus, and uncinate fasciculus was associated with go/no-go mean RT and flanker mean RT
Brain activation in regions of interest quantified using fNIRS Oxygenated Hemoglobin (HbO) during a go/no-go task of inhibitory control
Nz
Fp2h
Fp1h
AFp7
AFp8
AFp4
AFp3
AF5
AF6
AF2
AF1
AFF7
AFF8
AFF6
AFF5
F7h
F8h
F5h
F6h
F4h
F3h
FFT7
FFT8
FFC5
FFC6
FFC3
FFC4
FFC1
FFC2
FT8h
FT7h
FC5h
FC6h
FC3h
FC4h
FTT7
FTT8
FCC5
FCC6
T7h
T8h
CCP6
CCP5
TTP7
TTP8
TP7h
TP8h
LPA
RPA
Finding 3: R1 in the corona radiata and superior longitudinal fasciculus was associated with frontal task-related brain activation
